# Flow Cytometric Analysis of ZAP-70 Protein Expression for B-Cell Chronic Lymphocytic Leukemia Prognostication: Usefulness and Limitations

**DOI:** 10.7759/cureus.11691

**Published:** 2020-11-24

**Authors:** Muhammad Shariq Shaikh, Arsalan Ahmed, Summaiya Sohail, Amin Fahim, Sadiq Hussain Nohario, Shahid Pervez

**Affiliations:** 1 Pathology and Laboratory Medicine, Aga Khan University Hospital, Karachi, PAK; 2 Pathology, Isra University, Hyderabad, PAK; 3 Oncology, Nuclear Institute of Medicine and Radiotherapy, Jamshoro, PAK

**Keywords:** b-cell chronic lymphocytic leukemia (b-cll), zap-70, flow cytometry, pakistan

## Abstract

Objectives: The heterogenous clinical course in B-cell chronic lymphocytic leukemia (B-CLL) can be linked to several genetic and phenotypic characteristics of malignant B-cells. Prognostic analysis in B-CLL is routinely carried out to assist patient management; particularly to predict the time to initiate treatment. Increased ZAP-70 expression is a surrogate marker for unmutated immunoglobulin genes and inferior clinical outcomes which can be quantified to predict future outcomes in B-CLL patients. The study determined the ZAP-70 expression pattern using Z-index in Pakistani patients with B-CLL.

Methods: A retrospective analysis of B-CLL cases diagnosed and confirmed on flow cytometry at Aga Khan University Hospital for the last six years which had also undergone ZAP-70 analysis were included. In all these cases, ZAP-70 expression was quantified by measuring mean fluorescence intensities (MFIs) of normal B-cells, T-cells, and CLL-cells (CD19 and CD5 double-positive population). ZAP-70 expression was divided into high, low, and negative categories based on Z-index calculation. Mann-Whitney U test was utilized to determine the significance of ZAP-70 variations in different age groups and genders. P-value <0.05 was considered significant.

Results: A total of 120 patients of B-CLL had ZAP-70 analysis during the study period. The median age was 62 with an interquartile range of 35-87 and male to female ratio of 2:1. ZAP-70 expression was high in 18 (15%), low in 52 (43.3%) and negative in 50 (41.7%) cases. No significant difference in ZAP-70 expression with respect to the age or gender of the study population was identified using appropriate statistical calculations.

Conclusions: This study showed only 15% of B-CLL cases showing high ZAP-70 expression, a surrogate biomarker for possible aggressive behavior which may necessitate therapeutic intervention and close surveillance.

## Introduction

Chronic lymphocytic leukemia/small lymphocytic lymphoma (CLL/SLL) is a neoplasm characterized by progressive accumulation of small monomorphic mature B-lymphocytes that are functionally ineffective. In the absence of extramedullary tissue involvement (small lymphocytic lymphoma (SLL)), there must be ≥ 5 x 109/L monoclonal B-lymphocytes with a CLL phenotype. Accounting for around 25% of all leukemias in the United States, it is the most common type of leukemia in Western adults [1].

 The heterogeneity of CLL in terms of disease stage at diagnosis, cytogenetic abnormalities, biochemical and other markers is reflected in substantially different clinical outcomes among cases. Survival widely varies after diagnosis anywhere from 2-20 years with a median of 10 years [2,3]. This poses a great dilemma for treating physicians if and when to intervene. Prognostic bio-markers include lymphocyte doubling time, beta-2 microglobulin levels, expression of CD38, zeta chain associated (ZAP-70) proteins, immunoglobulin heavy-chain gene (IgHV) mutation status, and various other genetic abnormalities [4-6].

ZAP-70 is a tyrosine kinase normally required for T-cell differentiation and function. Normally, absent in B-lymphocytes, the increased expression in B-CLL is reported to be associated with poor prognosis [7]. Although discordance rates of up to 20% have been reported in various studies, the increased level of ZAP-70 is considered to be the most important discriminating gene between unmutated and mutated IgHV types of CLL. ZAP-70 possibly acts by decreasing the threshold for signaling through bcl2, hence facilitating its antiapoptotic effects [8].

ZAP-70 expression may be assessed by various methods including immunohistochemistry, however multiparameter flow cytometry (MFC) is a sophisticated technique that can readily be used to study various cellular characteristics simultaneously and objectively hence ZAP-70 expression analysis by MFC is a very useful rapid tool to determine prognosis in CLL. Mean fluorescence intensity (MFI) of anti-ZAP-70 labeled fluorochrome is calculated in CLL cells (CD19+, CD5+), normal B-cells (CD19+, CD5-), and T-cells (CD19-, CD5+). Relative intensities of expression in different cell subtypes can be used to establish positive and negative thresholds. Most studies done in the past have described ZAP-70 expression as positive or negative using distinct cut-offs [9]. A significant proportion of patients exist where ZAP-70 expression is in the gray zone. This cohort of patients must be identified using appropriate methodology and calculations and their clinical outcome correlated. This group of patients might explain the indeterminate prognostic group in whom the initial indolent course of B-CLL may abruptly transform into an aggressive phase and a rapid downhill course. Literature review revealed a lack of data focusing on low (gray zone) ZAP-70 expression in B-CLL. The current study is aimed at determining the pattern of ZAP-70 expression using Z-index in Pakistani B-CLL patients in order to have an insight about the proportion of gray zone (low expression) cases in addition to distinct positive and negative cases.

## Materials and methods

Study setting

The clinical laboratories of Aga Khan University Hospital (AKUH) serve as a referral center for the country. The laboratory is ISO 9001:2008 certified, accredited by Joint Commission International Accreditation (JCIA) and College of American Pathologist (CAP). The flow cytometry service caters for both in-patient and outside referrals with significant volumes. This retrospective analysis was performed at the Department of Pathology & Laboratory Medicine, AKUH. All B-CLL cases diagnosed and confirmed on flow cytometry for six consecutive years (2014-2019) on which ZAP-70 analysis was also done were included.

Confirmation of B-CLL on flow cytometry

In all cases, the diagnosis of B-CLL was confirmed immunophenotypically by flow cytometry. Gating was done on a bright CD45 positive lymphocyte population. The comprehensive panel of antibodies included: B-cell antigens (CD19, CD20, CD22, CD23, CD79a, FMC7), T-cell antigens (cCD3, CD3, CD5, CD7), myeloid antigens (MPO, CD13, CD33), markers of immaturity (CD34, Tdt), markers of clonality (kappa and lambda), and others (HLA-DR, CD117, CD10). For both B-CLL diagnosis and ZAP-70 expression analysis, FC500 MCL Flow Cytometer (Beckman Coulter, Miami, FL, USA) was used.

ZAP-70 expression analysis

Whole blood samples were collected in an EDTA tube and processed within 24 h after collection. Commercially prepared ZAP-70-phycoerythrin antibodies (Beckman Coulter Life Sciences) were used along with CD5-fluorescein isothiocyanate), CD19-phycoerythrin-cyanin 5.1, and CD3+CD56 phycoerythrin-cyanine 7. MFI of ZAP-70 was measured in normal B-cells (CD19+, CD5-), normal T-cells (CD3+, CD5+), and B-CLL cells (CD19+, CD5+). Z-index was calculated as follows:

Z-index = B-CLL MFI - B-cell MFI X 100 / T-cell MFI - B-cell MFI

As extensively optimized in a landmark study [10], the Z-index value of ≥ 27 was considered as high, ≤ 0.3 as negative, and low if falls between these two values.

Data analysis

Statistical Package for Social Science version 21 (IBM Corp., Armonk, NY, USA) was used for statistical analysis. Descriptive statistics were computed for all quantitative variables in terms of the median and interquartile range (IQR). The frequency with percentages was computed for qualitative characteristics. Shapiro-Wilk test was applied to determine the normality assumptions for all Z-index values. In case, normality assumption was not met, the Mann-Whitney U test was used. Mann-Whitney U test was also used to determine the significance of Z-index variation between different age groups and genders. The p-value <0.05 was taken as significance.

Ethical issues

An ethical exemption to conduct this analysis was granted by the Ethical Review Committee, Aga Khan University Hospital (#0404-290). Relevant counseling regarding the prognostic impact of the ZAP-70 results was provided to all who followed up later in the outpatient department.

## Results

A total of 120 B-CLL cases on which ZAP-70 expression was analyzed included 81 males and 39 females with an M:F=2:1. The median age of the study population was 62 years with an interquartile range of 35-87, it was 62 (40-87) years for males and 61 (35-75) years for females. All the cases had acceptable viability index above the cut-off of 70% therefore, all the cases were included for the analysis (mean ± SD viability was 94.43±5.21 %). ZAP-70 expression was high in 18 (15%), low in 52 (43.3%), and negative in 50 (41.7%) cases. Z-index value in males and females showed no significant difference (Table 1).

**Table 1:** Median and Inter Quartile Z-Index Values in Males and Females

Z-index	Males (n=81)		Females (n=39)		Total		P-value
	n (%)	Median [IQR]	n (%)	Median [IQR]	n (%)	Median [IQR]	
High (>27)	11 (13.6)	93.9 [62.3-142.9]	7 (17.9)	29.7 [27.6-93.0]	18 (15)	79.5 [29.7-112.4]	0.08
Low (0.4-27)	37 (45.7)	13.3 [10.1-21.2]	15 (38.4)	12.0 [9.2-18.8]	52 (43.3)	12.8 [9.8-20.8]	0.51
Negative (≤0.3)	33 (40.7)	2.7 [1.5-3.4]	17 (43.7)	2.0 [1.2-4.0]	50 (41.7)	2.5 [1.3-4.0]	0.84

Sixty-eight (56.7%) patients were above the age of 60 years. The Z-index analysis showed no significant difference in patients older than 60 years from patients ≤ 60 years of age (Table 2).

**Table 2:** Median and Inter Quartile Z-Index Values in ≤60 Years and > 60 Years Patients

Z-index	Age ≤ 60 years			Age > 60 years		Total	P-value
	n (%)	Median [IQR]	n (%)	Median [IQR]	n (%)	Median [IQR]	
High (>27)	10 (19.2)	93.5 [67.3-112.4]	8 (11.8)	44.9 [28.9-102.6]	18 (15)	79.5 [29.7-112.4]	0.23
Low (0.4-27)	24 (46.2)	11.9 [10.3-20.5]	28 (41.2)	13.4 [9.0-21]	52 (43.3)	12.8 [9.8-20.8]	0.95
Negative (≤0.3)	18 (34.6)	3.4 [1.3-4.1]	32 (47.1)	2.3 [1.3-3.4]	50 (41.7)	2.5 [1.3-4.0]	0.46

**Figure 1:**
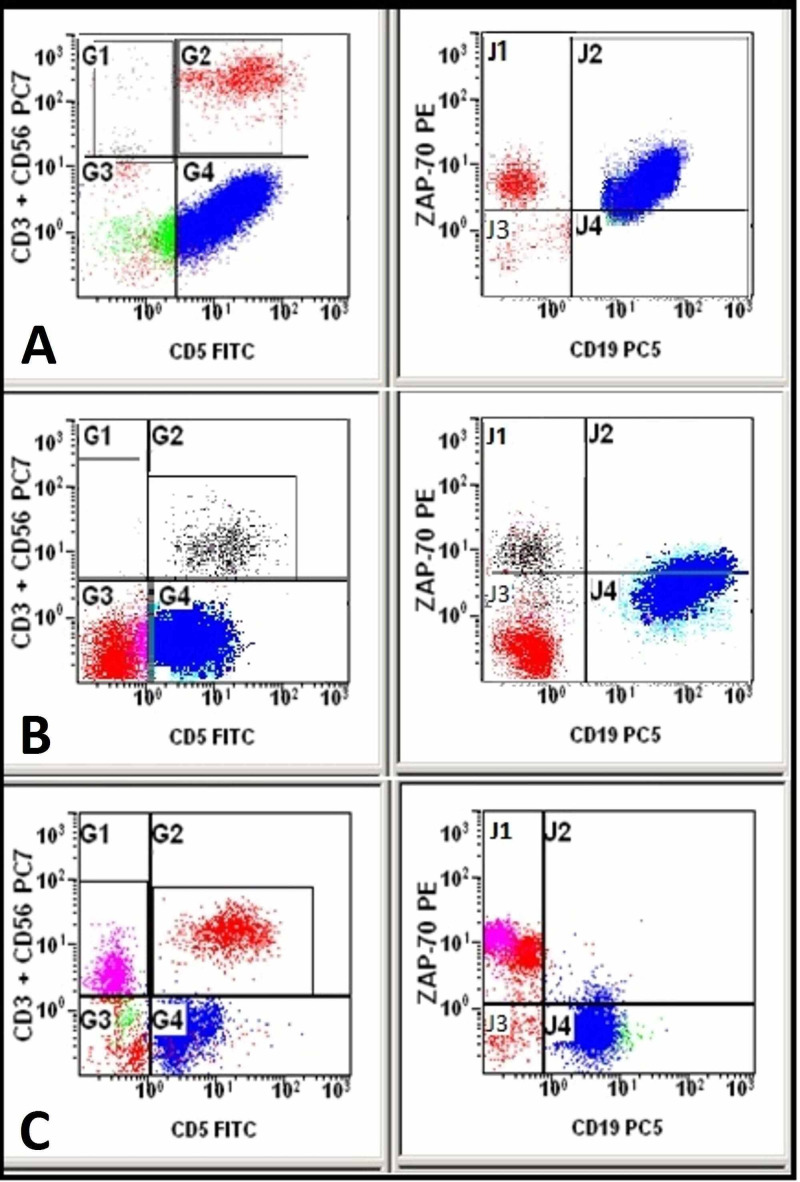
Dot plots showing normal B-cells (CD19+, CD5-) in green, T-cells (CD19-, CD5+, CD3+) in red, and CLL cells (CD19+, CD5+) in blue. ZAP-70 expression is high (A), low (B), and negative (C) in CLL cells (J2 quadrant)

Figure 1 shows cases with negative, low, and high ZAP-70 protein expression in B-CLL cases.

## Discussion

Chronic lymphocytic leukemia is a heterogeneous disease with widely variable outcomes. About one-third of the patients have an indolent disease with prolonged survival up to 20 years. The eventual cause of death in these patients is usually unrelated to CLL [11]. Some patients initially have a benign course extending over a period of five to 10 years followed by an aggressive phase. In its most aggressive form either following a benign course or upfront from the time of diagnosis, patients die within three to five years of diagnosis due to complications directly relatable to CLL [12,13].

The Rai and Binet staging systems take into account the degree of anemia, thrombocytopenia, lymphocytosis, lymph node involvement, and visceromegaly [14]. Both these systems are helpful for the clinical care of the patients and are in wide use for the past several years. However, considering the highly variable natural history of the disease, several laboratory parameters have also been described which include ZAP-70 and CD38 expression, beta-2 microglobulin levels, mutational status of IgHv, and other genetic abnormalities [4]. In most studies, ZAP-70 expression is reported as positive and negative, however, a large cohort lies in a gray zone with values as low as to be close to negative or as high as encroaching positive territory so called gray zone. This study provides information on ZAP-70 expression into three well-defined categories, high (positive), low (intermediate), and negative. The information is useful for the physician for treatment decisions and counseling patients right at the time of diagnosis.

B-CLL is twice as common in males than females; this fact was reflected exactly in our study as well with male to female ratio of 2:1 [15]. However, the median age at diagnosis in the current study was 62 years as compared to 72 years reported in western literature [11,16]. No statistically significant difference in Z-index was found with respect to the gender or age of the patients (Tables 1, 2).

A wide variation in ZAP-70 expression in B-CLL has been observed in studies done in various regions of the world. A previous Pakistani study reported a prevalence of 13.5% [17]. A prevalence of 25% was reported from India with very similar genetics and life-style similarities [9]. Both these studies used a cutoff of 20% for defining positive ZAP-70 expression. In some earlier analyses, ZAP-70 prevalence as high as 57% with a cut-off of 20% has also been described [18,19]. In the current analysis, a high Z-index was observed in 15% of patients with a cut-off of 27%. However, a significant proportion (41.6%) fell in the gray zone.

Although, the variation in the prevalence of ZAP-70 expression in different populations can be attributed to differences in genetics, ethnic origin, geographical and environmental factors, a large proportion of reports have used arbitrary threshold values to define ZAP-70 positivity [9,20]. In our study, we used Z-index to determine ZAP-70 expression rather than using simple quadrant analyses set by using isotype controls. Z-index incorporates ZAP-70 MFI signals in normal B-cells (negative control) in addition to T-cell MFI (positive control). This method is more sensitive and reproducible. Earlier studies have described ZAP-70 expression either as positive or negative using arbitrary cut-offs; mostly 20% [9]. This approach is likely to miss some cases with decreased ZAP-70 expression and hence important prognostic information. We adopted a Z-index strategy to quantify ZAP-70 from a landmark study for its merits [10]. In addition to standardized instrumentation setup and data analysis, unique sample fixation and permeabilization techniques have also been described in this study. The authors claim an 18- to 22-fold difference in the level of ZAP-70 protein measured in normal T-cells (ZAP-70 positive) as compared to normal B-cells (ZAP-70 negative). An evaluation of intra- and interlaboratory performance of this optimized assay in the analysis of CLL patient material has also been described in the analysis.

The advantages of ZAP-70 analysis as a predictor of prognosis in B-CLL include stability in its level over time. In one study with a median interval time of 25 months (4-179) in between sequential sampling, no changes in the pattern of ZAP-7- staining were observed in any case [20]. The flow cytometric analysis is a lot simpler, faster, and cheaper than PCR, sequencing, or western blotting which are used for IGHV mutation. As ZAP-70 is also expressed by T-lymphocytes and natural killer cells, careful separation of CLL B-cells is important. This can readily be achieved by appropriate labeling of lymphocyte-specific markers and appropriate gating strategies. Furthermore, due to its strong correlation, ZAP-70 expression analysis is an important surrogate marker for establishing IGHV mutational status particularly in developing countries where more sophisticated molecular techniques are unavailable.

The main limitation of our study is the lack of clinical follow-up primarily for the reason that a large number of specimens are outside referrals. However, the strength of the study is the use of Z-index rather than mere use of ZAP-70 percentage. Most other studies have utilized ZAP-70 percentages with arbitrary cut-offs. Using the Z-index strategy, our study highlights an important prognostic sub-category of “low or borderline or gray zone or indeterminate ZAP-70” expression in addition to distinct “high” and “negative” categories and it is precisely this significantly large group which need further research and insight.

## Conclusions

This study showed only 15% of B-CLL cases showing high ZAP-70 expression, a surrogate biomarker for possible aggressive behavior which may necessitate therapeutic intervention and close surveillance.
